# Chronic Antihyperglycemic Effect Exerted by Traditional Extracts of Three Mexican Medicinal Plants

**DOI:** 10.1155/2022/5970358

**Published:** 2022-11-09

**Authors:** Fernanda A. Espinoza-Hernández, Adolfo Andrade-Cetto

**Affiliations:** Laboratorio de Etnofarmacología, Facultad de Ciencias, Universidad Nacional Autónoma de México, Mexico City 04510, Mexico

## Abstract

Chronic hyperglycemia, the product of uncontrolled diabetes, leads to the appearance of vascular complications that can result in the premature death of diabetic patients. Consequently, pharmacological intervention with hypoglycemic agents could delay these complications and improve the quality of life of patients in the long term. Traditional Mexican medicine provides a great wealth of medicinal plants that are used for the treatment of type 2 diabetes, the most prevalent form of diabetes, accounting for nearly 90–95% of total cases. However, there is still a lack of studies that support their hypoglycemic effects, clarify their mechanisms of action, and report their long-term efficacy. Therefore, the aim of this study was to evaluate the chronic effects of the traditional extracts of some Mexican medicinal plants used by diabetic patients (*Ageratina petiolaris* (Moc. & Sessé ex DC.) R.M. King & H. Rob. (Asteraceae), *Calea urticifolia* (Mill.) DC. (Asteraceae), and *Eryngium cymosum* F.Delaroche (Apiaceae)) on hyperglycemia and hypertriglyceridemia. To achieve this goal, the aqueous extracts of these plants at their traditional doses were administered daily to streptozotocin-nicotinamide (STZ-NA) hyperglycemic Wistar rats for 42 days to assess their effects on nonfasting blood glucose (NFBG), glycated hemoglobin (HbA1c), and blood triglycerides (TG). The results showed that the *A. petiolaris* extract significantly reduced NFBG by 33% compared to its baseline (*p* = 0.0281). Besides, it prevented the increase in HbA1c by 2.63% (*p* = 0.0303) and diminished the AUC of TG (*p* = 0.0031) compared with the negative control. On the other hand, both *C. urticifolia* and *E. cymosum* prevented worsening of hyperglycemia by avoiding the significant increase in glucose levels seen in the negative control and the rise in HbA1c by 2.58% (*p* = 0.0156). These outcomes provide evidence for the first time of the antihyperglycemic effect of these Mexican medicinal plants, confirming their long-term efficacy in the control of chronic hyperglycemia.

## 1. Introduction

High blood glucose or hyperglycemia, the main feature of diabetes mellitus, is the primary cause of long-term complications and mortality in diabetic patients. This metabolic disease is a major factor in mortality worldwide since it was reported that 6.7 million adults between the ages of 20–79 died because of diabetes or its complications in 2021, i.e., 12.2% of global deaths from all causes in this age group [1]. Type 2 diabetes (T2D) is the most important form of diabetes, accounting for nearly 90–95% of all diabetes cases [2]; therefore, the growing global increase in diabetes is mainly due to the rise in T2D. In Mexico, diabetes became the third leading cause of death in 2020, surpassed only by COVID-19 and heart disease. According to the National Institute of Statistics and Geography (INEGI), of all deaths attributed to diabetes in that year, 98% were due to T2D [3].

T2D is characterized by hyperglycemia resulting from the dysfunction of pancreatic *β*-cells, normally in the context of insulin resistance, namely, a malfunction of this hormone in its target organs [2]. Usually, lifestyle changes and pharmacological therapy are recommended to delay long-term vascular complications and decrease cardiovascular risk [4]. Persistent hyperglycemia in poorly controlled patients leads to structural and functional alterations of the endothelium—which can progress to atherosclerosis—due to glucotoxicity processes, including the formation of advanced glycation end products (AGEs) and the activation of noncanonical pathways, such as polyols, that initially cause osmotic stress and finally damage cells by oxidative stress and abnormal stimulation of protein kinase C [5, 6].

Oxidative stress, defined as an overproduction of reactive oxygen species (ROS), is also associated to pancreatic *β* cell dysfunction and diabetic end-organ damage in untreated diabetic patients, such as chronic kidney disease, retinopathies, heart attack, and stroke [7]. Besides, it has been reported that pulmonary defects, hepatic dysfunction, and sexual dysfunction are long-term complications of diabetes because of oxidative stress [8–10]. Although ROS has many physiological roles, such as cell proliferation and angiogenesis, vasodilation, hormone synthesis, insulin secretion, and insulin sensitivity, excessive amounts disrupt oxidation-reduction balance, impairing cellular function and altering cell membrane structures [7, 11, 12]. In this regard, medicinal plants have become relevant in recent years since they are an important source of antioxidants and antiinflammatory molecules whose properties make them capable of neutralizing ROS to generate stable compounds [13].

Patients with diabetes who live in developing countries like Mexico have a strong idiosyncrasy in the use of medicinal plants; in addition, there is a lack of adequate institutional health services, so they resort to the use of traditional and complementary medicine. Currently, it is estimated that approximately 88% of the world's population uses this type of medicine as part of their primary care, mainly due to its low cost and cultural background [14]. Specifically, it is calculated that at least 800 plants are used for the treatment of diabetes in Mexico [15]. However, for most of them, there are no studies that support their pharmacological effect, or studies on their mechanisms of action are still limited. In this context, it is imperative to also assess the efficacy of these alternative treatments in the long term.

Previously, our work group documented the acute hypoglycemic effect and the phytochemical content of *Ageratina petiolaris* (Moc. & Sessé ex DC.) R.M. King & H. Rob. (Asteraceae) [16], *Calea urticifolia* (Mill.) DC. (Asteraceae) [17], and *Eryngium cymosum* F. Delaroche (Apiaceae) [18, 19]. These species are part of the traditional Mexican medicine for the treatment of T2D and are used by diabetic patients in the states of Mexico and Hidalgo. It was reported that these plants are consumed as tea throughout the day as the so-called “agua de uso.” Although their efficacy in the short term has already been proven, their chronic effect has not yet been determined. Therefore, the aim of this study was to investigate the impact of these plant extracts at their traditional doses on hyperglycemia, particularly on glycated hemoglobin (HbA1c), the gold standard used to evaluate the glycemic control of patients with T2D in clinical practice. As a secondary objective, the chronic effect of these plants on hypertriglyceridemia was also evaluated.

## 2. Materials and Methods

### 2.1. Chemicals

Nicotinamide (NA; N0636) and streptozotocin (STZ; S0130) were purchased from Sigma-Aldrich (Steinheim, Germany).

### 2.2. Plant Material and Preparation of Extracts

The aerial parts of *A. petiolaris* were collected in Tenancingo, State of Mexico, while the aerial parts of *C. urticifolia* and *E. cymosum* were collected in Tamala and Huejutla de Reyes, Hidalgo, respectively, in June 2019. After collection, the plant material was dried and ground for further processing. Afterwards, the aqueous extract, equivalent to the traditionally consumed tea, of each plant was prepared by stirring 20 g of ground aerial parts in 500 ml of boiling distilled water for 15 min. Then, the mixtures were filtered, deep-frozen at −40°C, and lyophilized to obtain the final extract.

The phytochemical composition of the same extracts is already reported in the literature by our group. We used the previously reported doses that demonstrated an acute hypoglycemic effect to perform the current chronic experiment [16–19].

### 2.3. Experimental Animals and Bioethical Considerations

Eight-week-old male and female Wistar rats were obtained from the animal facility of the School of Sciences, UNAM, and maintained under controlled conditions (12 h light-dark cycle period, 25°C, and 55% humidity). The animals were fed standard rodent chow, containing 49% carbohydrate, 23% protein, 3% fat (Agribrands Purina Mexico, S. de R.L de C.V., Mexico) and given water *ad libitum*. All procedures and handling performed on the animals were approved by the Academic Ethics and Scientific Responsibility Commission (CEARC) of the School of Sciences, UNAM (approved protocols: T_2019_09_004 and T_2019_09_006) and carried out according to the Guide for the Care and Use of Laboratory Animals [20].

### 2.4. Induction of Hyperglycemia

As previously reported, hyperglycemia was induced overnight in fasted rats [21]. In brief, NA, dissolved in physiological solution, was injected intraperitoneally at a dose of 150 mg/kg b.w. and, 15 minutes later, STZ, prepared in acetate buffer 0.1 M and pH 4.5, was administered intravenously at a dose of 65 mg/kg b.w. One week later, animals with frank polydipsia, polyuria, polyphagia, and nonfasting blood glucose (NFBG) levels above 300 mg/dl were considered for the study.

### 2.5. Experimental Design

The rats were randomly assigned into 6 groups (*n* = 6 each):

  Group 1: a normoglycemic control, not induced with the STZ-NA model to assess the effectiveness of hyperglycemia induction, which received a physiological solution (vehicle)
  Group 2: a negative hyperglycemic control was administered with a physiological solution (vehicle)
  Group 3: a positive hyperglycemic control, which received metformin-glibenclamide (Aurax®) at doses of 500 mg/kg b.w. and 5 mg/kg b.w. [22], respectively
  Group 4: an experimental hyperglycemic group which was administered the *A. petiolaris* extract (160 mg/kg b.w.) [16]
  Group 5: an experimental hyperglycemic group, which was administered the *C. urticifolia* extract (41 mg/kg b.w.) [17]
  Group 6: an experimental hyperglycemic group that received the *E. cymosum* extract (47 mg/kg b.w.) [18]

**Figure 1:**
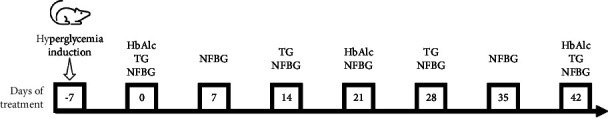
Experimental design. NFBG: nonfasting blood glucose; TG: blood triglycerides; and HbA1c: glycated hemoglobin.

All treatments were given by gavage daily for 42 days. NFBG was monitored weekly, while HbA1c was measured on day 0, day 21, and day 42. Blood triglycerides (TG) were determined biweekly (Figure 1).

### 2.6. Quantification of Biochemical Parameters

NFBG was measured using an Accu-Chek® Active glucometer. HbA1c was determined using a DCA Vantage® analyzer. TGs were quantified using an Accutrend® Plus system. All blood samples were obtained from the tail vein and measures were done in duplicate.

### 2.7. Statistical Analysis

All graphics and analysis were done in GraphPad Prism version 9.2.0, GraphPad Software, San Diego, California, USA (https://graphpad.com). The data are represented as the mean ± standard error of the mean (SEM). Areas under the curves (AUC) were calculated from the original time courses. To compare the final values of the biochemical parameters with their initial values, paired Student's *t*-tests were applied. Ordinary one-way ANOVA and Tukey's post-*hoc* tests were performed to compare means among groups, while repeated measures ANOVA and Dunnett's post-*hoc* tests were carried out to compare means with their baseline. *P* values less than 0.05 were considered statistically significant.

## 3. Results and Discussion

### 3.1. General Overview

According to the summary of the results presented in Table 1, hyperglycemia was effectively induced in the animals, as all hyperglycemic groups exhibited significantly higher initial NFBG levels than the normoglycemic group (*p* < 0.05). Likewise, the hyperglycemic organisms showed higher HbA1c levels at the beginning of the experiment (*p* < 0.05). Nevertheless, TG did not change one week after the STZ-NA induction. At the end of the experimental period, the biochemical parameters of the normoglycemic control were not modified, while those of the negative hyperglycemic control significantly increased compared with their baseline (*p* < 0.001), indicating a worsening of the hyperglycemic condition.

On the other hand, the positive hyperglycemic control exhibited a significant reduction in all parameters compared with those observed in the negative control (NFBG: *p* = 0.0281; HbA1c: *p* = 0.0131; TG: *p* = 0.0130), showing that metformin-glibenclamide administration prevented the worsening of hyperglycemia and hypertriglyceridemia. Similarly, *A. petiolaris* extract showed the same behavior in all analyzed parameters (NFBG: *p* = 0.0303; HbA1c: *p* = 0.0303; and TG: *p* = 0.0105).

Regarding *C. urticifolia* and *E. cymosum*, both plant extracts behaved in a similar way by preventing a significant increase in NFBG. Even though they did not have a hypoglycemic effect, both showed a sustained antihyperglycemic effect that did not reach significant differences compared with the negative control due to the high dispersion of the data (*C. urticifolia* NFBG: *p* = 0.2349 and *E. cymosum* NFBG: *p* = 0.2638). However, their effectiveness on hyperglycemia can be corroborated by noting the HbA1c levels, which only increased by 2.42% (*C. urticifolia* vs. negative control: *p* = 0.0156) and 2.98% (*E. cymosum* vs. negative control: *p* = 0.0156). Despite their antihyperglycemic effects, both plant extracts were less effective in controlling TG.

### 3.2. Effect of Plant Extracts on Hyperglycemia

To prevent or delay diabetic complications, it is recommended that glucose levels be maintained within specific ranges, so reaching the glycemic targets proposed by health organizations is the principal goal of any therapy applied to diabetic patients [23]. According to the American Diabetes Association (ADA), good glycemic control involves an assessment of HbA1c, continuous glucose monitoring through a device that measures glucose levels throughout the day, and blood glucose monitoring, which refers to specific measures in fasting and/or nonfasting [24]. Specifically, it has been reported that glucose measurements taken after meals, i.e., NFBGs, are a better marker of glycemic control than fasting glucose values in diabetic patients [25] and animal models [26], showing a positive correlation with HbA1c levels. Thus, NFBG was selected for this study as a suitable indicator of treatment efficacy.

**Figure 2:**
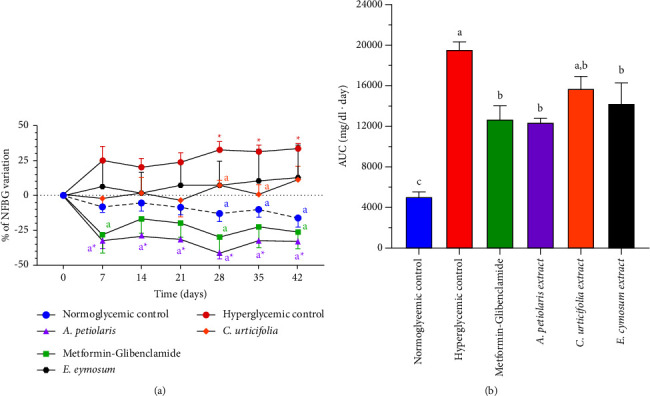
Effect of plant extracts on NFBG. (a) Percentage variation of NFBG. Hyperglycemia exhibited by negative control increased by 34% on day 42, while positive control and *A. petiolaris* extract decreased hyperglycemia by 26% and 33%, respectively. Both *C. urticifolia* and *E. cymosum* extracts prevent the worsening of the hyperglycemic state. ‘a' vs. negative hyperglycemic control in that time, ^*∗*^ vs. baseline; *p* < 0.05; *n* = 6. (b) AUC of original time courses of NFBG. All therapeutic treatments diminished AUC against negative control. Different letters over the bars indicate statistically significant differences; *p* < 0.05; *n* = 6.

As shown in Figure 2(a), the NFBG time course of the negative control was the highest and increased with time. The progression of the worsening of the hyperglycemic condition displayed by the negative control could be due to glucotoxicity, whose biochemical mechanisms lead to the generation of chronic oxidative stress that ultimately impair *β*-cell function [27]. ROS overproduction caused by high intracellular glucose activates assorted biochemical pathways: hyperglycemia causes accumulation of electrons that generates free radicals in mitochondria due to partial inhibition of electron transport in complex III and it is promoted the formation of H_2_O_2_ and *α*-ketoaldehyde because of the auto-oxidation of the accumulated glyceraldehyde-3-phosphate at high glucose concentration. Similarly, abnormal PKC activation increased intracellular AGEs formation and angiotensin II production, and augmented polyol and hexosamine pathway fluxes also play an important role in free radical generation by hyperglycemia [6]. A vicious cycle is observed since continuous hyperglycemia provokes *β*-cell damage that results in higher blood glucose levels due to the impairment of both glucose-induced insulin secretion and insulin gene expression. Moreover, it has been recently reported that persistent hyperglycemia promoted by the STZ-NA induction could contribute to the development of diabetic complications through an increased activity of aldose reductase, an enzyme involved in the progression of cataracts and diabetic retinopathy [28].

Usually, T2D is diagnosed when symptoms appear, namely, when the disease is already in advanced stages. People in rural communities receive their diagnosis from medical services, which guide patients towards allopathic treatment. At this point, diabetic patients start to consume plants instead of conventional medicine or in addition to the prescribed medication because of their cultural traditions. In this regard, the research question is whether medicinal plants can control hyperglycemia in this stage. Although the intervention with plant extracts promoted lower NFBG time courses, two different behaviors were appreciated. *A. petiolaris* (160 mg/kg b.w.) diminished glucose levels by ∼200 mg/dl during the first week of treatment and subsequently showed an antihyperglycemic effect that, overall, resulted in a significant reduction in its AUC compared with the negative control (*p* = 0.0022; Figure 2(b)). On the other hand, both *C. urticifolia* (41 mg/kg b.w.) and *E. cymosum* (47 mg/kg b.w.) displayed a chronic antihyperglycemic effect from the first week of treatment, resulting in lower AUCs. However, only the AUC of *E. cymosum* reached a statistical difference compared with the negative control (*p* = 0.0352). These results suggest that these plants with proven acute hypoglycemic effects can manage chronic hyperglycemia by improving glucose homeostasis at their traditional doses.

In addition to reducing blood glucose levels by regulating glucose homeostasis, medicinal plants and their isolated compounds have been shown to manage diabetic complications by directly counteracting oxidative stress. To exemplify, berberine from the rhizome of *Coptis chinensis* Franch (Ranunculaceae) and cortex of *Phellodendron amurense* Rupr. (Rutaceae) controlled blood glucose levels by decreasing oxidative stress and suppressing the polyol pathway in STZ rats [6]. Similarly, the potential antioxidant effect of *E. planum* L. was corroborated in an *in vitro* investigation, resulting in a considerable scavenging effect against free radicals. In the same work, this species diminished blood glucose levels acutely in alloxan-induced diabetic mice [29]. In another study, *E. carlinae* F. Delaroche was demonstrated to reduce hyperglycemia, renal lipid accumulation, and oxidative stress in rats fed a high-fat and high-fructose diet, suggesting its therapeutic effect on renal dysfunction [30]. Based on these studies, the plant extracts tested here may control hyperglycemia by reducing oxidative stress, which could have a positive effect on the management of long-term complications of diabetes. Thus, it is encouraged to assess these mechanisms of action in further studies.

As shown in Figure 2, the administration of metformin (500 mg/kg b.w.) and glibenclamide (5 mg/kg b.w.) promoted effects on NFBG like those shown by the *A. petiolaris* extract, namely, a hypoglycemic effect in the first week and a subsequent chronic antihyperglycemic effect, producing a significantly different AUC than that exhibited by the negative control (*p* = 0.0033). It has been documented in clinical studies that the combination of both drugs produces a different pharmacokinetic profile than when they are administered separately, reducing fasting blood glucose, NFBG, and HbA1c levels more effectively [23]. Therefore, combination therapy treating multiple pathophysiological defects of diabetes can exert superior control of hyperglycemia, in this case, by providing a suppressor of hepatic glucose production and a systemic insulin sensitizer (metformin) [31] that promotes better functionality of the insulin stimulated by glibenclamide [32].

Similar to metformin, it has been reported by our research group that *A. petiolaris*, *C. urticifolia*, and *E. cymosum* can reduce hepatic glucose production by inhibiting the activity of some of the participating step-limiting enzymes [17, 18, 33]. However, the differential effects on glucose levels exerted by the extracts in the present study could be explained either by a synergistic effect with other unknown mechanisms of action involved in each of them or by the traditional doses used.

According to the previous phytochemical analyses, chlorogenic acid is one of the major compounds present in the aqueous (traditional) extract of these three medicinal plants [16, 17, 19]. This phenolic compound has been proven to have an assorted mechanism of action on glucose metabolism, as it prevents glucose release by inhibiting the activity and expression of glucose-6-phosphatase, reduces glucose absorption, and enhances insulin functionality by promoting the phosphorylation of AMP-activated protein kinase [34]. Moreover, it has been reported that this phenolic acid can be used to prevent and treat diabetic complications by reducing oxidative stress [35].

On the other hand, rutin and rosmarinic acid, two of the main compounds of *C. urticifolia* and *E. cymosum* aqueous extracts [17, 19], respectively, have been proven to decrease carbohydrate absorption from the small intestine, protect *β* cells against degeneration, enhance glucose uptake, inhibit hepatic gluconeogenesis, and reduce diabetic complications [36, 37]. In this regard, the phytochemical content of the traditional extracts of these plants may explain the control exerted on hyperglycemia in the current study.

**Figure 3:**
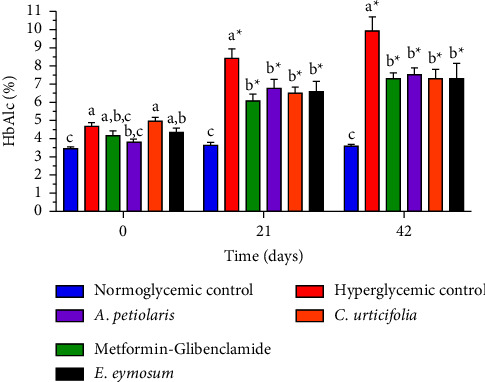
Effect of plant extracts on HbA1c. Chronic administration of treatments prevented the elevation of HbA1c through the experimental period. Different letters over the bars indicate statistically significant differences at each time, ^*∗*^vs. the initial time; *p* < 0.05; *n* = 6.

The chronic antihyperglycemic effect exhibited by the plant extracts resulted in a significant reduction in HbA1c levels compared with the negative control and their initial values after day 21 (*p* < 0.05; Figure 3). In the untreated hyperglycemic rats, HbA1c increased progressively to almost 10%, while rats treated with the plant extracts only augmented to ∼7.5%. It has been reported that fasting hyperglycemia is the principal contributor to overall hyperglycemia in poorly controlled diabetic patients, i.e., patients with HbA1c levels >8.5%, while postprandial hyperglycemia has a more relevant role in moderately well-controlled patients, i.e., <7.3% [38]. Hence, it could be inferred that plant extracts may impact HbA1c by decreasing fasting hyperglycemia, in which the principal contributor is hepatic glucose production [39]. As mentioned earlier, *A. petiolaris*, *C. urticifolia*, and *E. cymosum* exert significant control over this mechanism of action.

According to the ADA, achieving <7% of HbA1c is the recommended glycemic target to avoid complications. However, less stringent targets, i.e., <8%, maybe encouraged if the benefits of an intensive goal cannot be accomplished due to the patient's life expectancy [24]. In this context, the plants tested here exhibited a good control of HbA1c since they maintained this parameter below 8%. These results suggest that diabetic patients can clinically control overall hyperglycemia by consuming these medicinal plants. In fact, the HbA1c reduction values exhibited by the three plant extracts ranged from 2.4% to 3.7%, whose efficacy has only been compared with the administration of medicinal plants or herbal formulas at high doses. For instance, chronic treatment with Diabac, a polyherbal formulation, for 28 days in STZ-NA hyperglycemic rats reduced HbA1c to 7.18% using the highest dose of 1000 mg/kg [40]. Likewise, the Indian medicinal plant *Tribulus terrestris* L. (Zygophyllaceae) showed only a 2.25% total decrease in HbA1c after daily administration of the highest dose of 300 mg/kg for 16 weeks to STZ-NA hyperglycemic rats [28]. Considering these outcomes, it could be considered the possible clinical application of these Mexican medicinal plants as phytomedicines.

### 3.3. Effect of Plant Extracts on Hypertriglyceridemia

In addition to hyperglycemia, altered lipid metabolism plays an essential role in the development of diabetic complications [41]. It is widely recognized and documented that dyslipidemia is a major contributor to the progression of both T1D and T2D as well as diabetic complications, i.e., high levels of lipid-rich lipoproteins, such as very low-density lipoprotein (VLDL) and low-density lipoprotein (LDL) [42]. In paticular, there is an overproduction and a deficient clearance of triglyceride-rich lipoproteins and their remnants due to the deficient activity of lipoprotein lipase, which is regulated by insulin, and impaired removal of chylomicrons and VLDL by the liver. These abnormalities are a consequence of insulin resistance in the liver and small intestine in T2D, which together leads to fasting and postprandial hypertriglyceridemia and the development of a highly atherogenic lipid profile [43].

**Figure 4:**
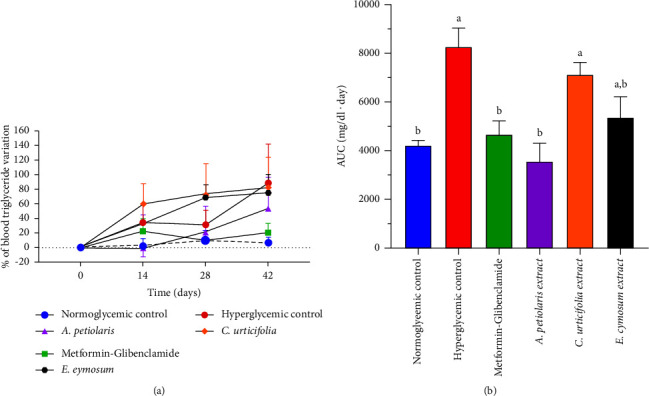
Effect of plant extracts on TG. (a) Percentage variation of TG. It is observed an increase in this metabolic parameter in negative control (89%), *C. urtificifolia* group (83%), and *E. cymosum* group (76%) since day 14. Positive control and *A. petiolaris* extract prevented the elevation of TG. (b) AUC of original time courses of TG. Only metformin-glibenclamide and *A. petiolaris* extract diminished AUC against negative control. Different letters over the bars indicate statistically significant differences; *p* < 0.05; *n* = 6.

In the current study, TGs were measured as a marker of the possible antilipidemic activity of the plant extracts. However, only *A. petiolaris* significantly prevented the increase in this metabolic parameter, leading to a significant reduction in its AUC (*p* = 0.0031; Figure 4). This antihypertriglyceridemic effect could be attributed to L-*chiro*-inositol, one of the main components of *A. petiolaris* aqueous extract [16], since it has been reported that other inositol isoforms, such as myo-inositol and D-*chiro*-inositol, have beneficial effects on lipid profiles by lowering triglyceride, LDL, and VLDL levels in different clinical trials [44–46]. Furthermore, D-*chiro*-inositol-enhanced hepatic insulin sensitivity in STZ-induced rats fed a high-fat diet by upregulating components of the insulin signaling pathway [47]. Therefore, it is suggested to analyze the impact of the *A. petiolaris* extract and L-*chiro*-inositol on the metabolism of specific lipid particles, such as VLDL and LDL, as well as high-density lipoproteins, in further studies.

## 4. Conclusions

The present work provides evidence on the effect of traditionally used extracts of *A. petiolaris*, *C. urticifolia*, and *E. cymosum* on chronic hyperglycemia, being the first report on their long-term efficacy and their potential to delay diabetic complications. These plants, which are a part of traditional Mexican medicine, were shown to significantly decrease HbA1c compared with the untreated hyperglycemic group, displaying an antihyperglycemic effect throughout the study. Moreover, only *A. petiolaris* could effectively reduce TG, which demonstrates its possible antilipidemic effect. Since at this time we were not able to measure more parameters and provide more analysis, such as histopathology of the pancreas or *in vitro* tests, further studies are recommended to assess the long-term impact of the plant extracts on *β*-cell architecture, insulin levels, specific lipid particles, and oxidative stress-related pathways. However, it is expected that the results obtained in this study establish the bases to understand the chronic effect of these Mexican plants and contribute to the development of phytomedicines for the treatment of T2D.

## Figures and Tables

**Table 1:** General effect of plant extracts on biochemical parameters.

	Nonfasting blood glucose (mg/dl)			Glycated hemoglobin (%)			Blood triglycerides (mg/dl)		
	Initial	Final	Delta	Initial	Final	Delta	Initial	Final	Delta
Normoglycemic control	131 ± 7	112 ± 14	**−19**	3.47 ± 0.08	3.65 ± 0.03	**0.18**	101 ± 8	105 ± 5	**4**
Negative hyperglycemic control	375 ± 23^a^	500 ± 25^a^^*∗*^	**125**	4.72 ± 0.18^a^	9.95 ± 0.75^a^^*∗*^	**5.23**	146 ± 24	244 ± 46^a^	**98**
Positive hyperglycemic control (metformin-glibenclamide)	392 ± 21^a^	284 ± 42^b^	**−108**	4.18 ± 0.27	7.32 ± 0.31^b^^*∗*^	**3.13**	95 ± 7	116 ± 18^b^	**21**
Experimental hyperglycemic group 1 (*A. petiolaris* extract)	425 ± 10^a^	286 ± 25^b^^*∗*^	**−139**	3.83 ± 0.11	7.57 ± 0.34^b^^*∗*^	**3.73**	88 ± 15	113 ± 17^b^	**25**
Experimental hyperglycemic group 2 (*C. urticifolia* extract)	366 ± 16^a^	414 ± 48	**48**	4.95 ± 0.25^a^	7.37 ± 0.44^b^^*∗*^	**2.42**	125 ± 14	203 ± 24	**78**
Experimental hyperglycemic group 3 (*E. cymosum* extract)	322 ± 13^a^	355 ± 71	**33**	4.38 ± 0.18^a^	7.37 ± 0.80^b^^*∗*^	**2.98**	101 ± 16	166 ± 21^*∗*^	**65**

In the same column,^**a**^vs. normoglycemic control,^**b**^vs. negative hyperglycemic control; *p* < 0.05, *n* = 6. In the same row:^*∗*^vs. the initial value;*p* < 0.05, *n* = 6.

## Data Availability

The data used to support the findings of this study are included within the article.

## References

[1] International Diabetes Federation (2021). *IDF Diabetes Atlas*.

[2] American Diabetes Association (2022). Classification and diagnosis of diabetes: standards of medical care in diabetes—2022. *Diabetes Care*.

[3] Instituto Nacional de Estadística y Geografía (2021). *Estadísticas a propósito del día mundial de la diabetes (14 de noviembre). Comunicado de prensa núm. 645/21*.

[4] American Diabetes Association (2022). Prevention or delay of type 2 diabetes: standards of medical care in diabetes — 2022. *Diabetes Care*.

[5] Garg S. S., Gupta J. (2022). Polyol pathway and redox balance in diabetes. *Pharmacological Research*.

[6] Panigrahy S. K., Bhatt R., Kumar A. (Feb. 2017). Reactive oxygen species: sources, consequences and targeted therapy in type 2 diabetes. *Journal of Drug Targeting*.

[7] Elbatreek M. H., Pachado M. P., Cuadrado A., Jandeleit-Dahm K., Schmidt H. H. H. W. (May 2019). Reactive oxygen comes of age: mechanism-based therapy of diabetic end-organ damage. *Trends in Endocrinology & Metabolism*.

[8] Kumar A., Bharti S. K., Kumar A. (Sep. 2014). Type 2 diabetes mellitus: the concerned complications and target organs. *Apollo Medicine*.

[9] Shokri F., Shokoohi M., Niazkar H. R. (Jan. 2019). Investigation the spermatogenesis and testis structure in diabetic rats after treatment with galega officinalis extract. *CRESCENT Journal Medicine Biology Science*.

[10] Shoorei H., Khaki A., Shokoohi M. (2020). Evaluation of carvacrol on pituitary and sexual hormones and their receptors in the testicle of male diabetic rats. *Human & Experimental Toxicology*.

[11] Shokoohi M., Farashah M. S. G., Khaki A. (Jul. 2019). Protective effect of fumaria parviflora extract on oxidative stress and testis tissue damage in diabetic rats. *CRESCENT Journal of Medical Biological Science*.

[12] Shoorei H., Khaki A., Khaki A. A., Hemmati A. A., Moghimian M., Shokoohi M. (2018). The ameliorative effect of carvacrol on oxidative stress and germ cell apoptosis in testicular tissue of adult diabetic rats. *Biomedicine & Pharmacotherapy*.

[13] Sharma P., Hajam Y. A., Kumar R., Rai S. (2022). Complementary and alternative medicine for the treatment of diabetes and associated complications: a review on therapeutic role of polyphenols. *Phytomedicine*.

[14] World Health Organization (2019). *WHO Global Report on Traditional and Complementary Medicine 2019*.

[15] Escandón-Rivera S. M., Mata R., Andrade-Cetto A. (2020). Molecules isolated from mexican hypoglycemic plants: a review. *Molecules*.

[16] Bustos-Brito C., Andrade-Cetto A., Giraldo-Aguirre J. D., Moreno-Vargas A. D., Quijano L. (Mar. 2016). Acute hypoglycemic effect and phytochemical composition of *Ageratina petiolaris*. *Journal of Ethnopharmacology*.

[17] Andrade-Cetto A., Espinoza-Hernández F., Mata-Torres G. (Jan. 2021). Hypoglycemic effect of *Calea urticifolia* (mill.) DC. *Evidence-based Complementary and Alternative Medicine*.

[18] Espinoza-Hernández F., Andrade-Cetto A., Escandón-Rivera S., Mata-Torres G., Mata R. (2021). Contribution of fasting and postprandial glucose-lowering mechanisms to the acute hypoglycemic effect of traditionally used *Eryngium cymosum* F.Delaroche. *Journal of Ethnopharmacology*.

[19] Romo-Pérez A., Escandón-Rivera S. M., Miranda L. D., Andrade-Cetto A. (Apr. 2022). Phytochemical study of *Eryngium cymosum* F. Delaroche and the inhibitory capacity of its main compounds on two glucose-producing pathway enzymes. *Plants*.

[20] (2011). *National Research Council (US) and Committee for the Update of the Guide for the Care and Use of Laboratory Animals, Guide for the Care and Use of Laboratory Animals*.

[21] Masiello P., Broca C., Gross R. (1998). Experimental NIDDM: development of a new model in adult rats administered streptozotocin and nicotinamide. *Diabetes*.

[22] Alzain S. D., Eltahir Mudawi M. M., Mohamed A. W. H., Imran M., Alhassan Attaalfadeel H. M. (Jul. 2021). Effect of metformin, Glibenclamide, Sitagliptin and their combinations on male rats fertility. *Journal of Young Pharmacists*.

[23] Abrahamson M. J. (2004). Optimal glycemic control in type 2 diabetes mellitus. *Archives of Internal Medicine*.

[24] American Diabetes Association (2022). 6 glycemic targets: standards of medical care in diabetes—2022,” diabetes care.

[25] Avignon A., Radauceanu A., Monnier L. (1997). Nonfasting plasma glucose is a better marker of diabetic control than fasting plasma glucose in type 2 diabetes. *Diabetes Care*.

[26] Islam M. S. (2011). Fasting blood glucose and diagnosis of type 2 diabetes. *Diabetes Research and Clinical Practice*.

[27] Fu J., Cui Q., Yang B. (2017). The impairment of glucose-stimulated insulin secretion in pancreatic *β*-cells caused by prolonged glucotoxicity and lipotoxicity is associated with elevated adaptive antioxidant response. *Food and Chemical Toxicology*.

[28] Vangalapati B., Manjrekar P. A., Hegde A. (May 2020). Antidiabetic and aldose reductase inhibitory potentials of land caltrops aqueous extract in streptozotocin-nicotinamide induced diabetic rats. *Journal of Herbmedicine Pharmacology*.

[29] Sadiq A., Rashid U., Ahmad S. (2020). Treating hyperglycemia from *Eryngium caeruleum* M. Bieb: in-vitro*α*-glucosidase, antioxidant, in-vivo antidiabetic and molecular docking-based approaches. *Frontiers of Chemistry*.

[30] Pérez-Ramírez I. F., Enciso-Moreno J. A., Guevara-González R. G., Gallegos-Corona M. A., Loarca-Pina G., Reynoso-Camacho R. (Jan. 2016). Modulation of renal dysfunction by smilax cordifolia and eryngium carlinae , and their effect on kidney proteome in obese rats. *Journal of Functional Foods*.

[31] He L. (Nov. 2020). Metformin and systemic metabolism. *Trends in Pharmacological Sciences*.

[32] Lorenzati B., Zucco C., Miglietta S., Lamberti F., Bruno G. (2010). Oral hypoglycemic drugs: pathophysiological basis of their mechanism of actionoral hypoglycemic drugs: pathophysiological basis of their mechanism of action. *Pharmaceuticals*.

[33] Mata-Torres G., Andrade-Cetto A., Espinoza-Hernández F. A., Cárdenas-Vázquez R. (2020). Hepatic glucose output inhibition by mexican plants used in the treatment of type 2 diabetes. *Frontiers in Pharmacology*.

[34] Naveed M., Hejazi V., Abbas M. (2018). Chlorogenic acid (CGA): a pharmacological review and call for further research. *Biomedicine & Pharmacotherapy*.

[35] Yan Y., Zhou X., Guo K., Zhou F., Yang H. (2020). Use of chlorogenic acid against diabetes mellitus and its complications. *Journal of Immunology Research*.

[36] Ghorbani A. (2017). Mechanisms of antidiabetic effects of flavonoid rutin. *Biomedicine & Pharmacotherapy*.

[37] Ngo Y. L., Lau C. H., Chua L. S. (2018). Review on rosmarinic acid extraction, fractionation and its anti-diabetic potential. *Food Chemistry Toxicology*.

[38] Monnier L., Lapinski H., Colette C. (2003). Contributions of fasting and postprandial plasma glucose increments to the overall diurnal hyperglycemia of type 2 diabetic patients. *Diabetes Care*.

[39] Rizza R. A. (2010). Pathogenesis of fasting and postprandial hyperglycemia in type 2 diabetes: implications for therapy. *Diabetes*.

[40] Agrawa R., Maheshwari R., Balaraman R., Seth A. (Jul. 2015). Anti-hyperglycemic and anti-lipidemic activities of diabac (a polyherbal formulation) in streptozotocin-nicotinamide induced type 2 diabetic rats. *Pharmacognosy Journal*.

[41] Kalra S., Unnikrishnan A. G., Baruah M. P., Sahay R., Bantwal G. (2021). Metabolic and energy imbalance in dysglycemia-based chronic disease. *Diabetes, Metabolic Syndrome and Obesity: Targets and Therapy*.

[42] Busik J. V. (2021). Lipid metabolism dysregulation in diabetic retinopathy. *Journal of Lipid Research*.

[43] Adeli K., Xiao C., Higgins V., Ridgway N. D., McLeod R. S. (2021). Diabetic dyslipidaemia. *Biochemistry of Lipids, Lipoproteins and Membranes*.

[44] Caputo M., Bona E., Leone I. (May 2020). Inositols and metabolic disorders: from farm to bedside. *Journal of Traditional and Complementary Medicine*.

[45] Shokrpour M., Foroozanfard F., Afshar Ebrahimi F. (2019). Comparison of myo-inositol and metformin on glycemic control, lipid profiles, and gene expression related to insulin and lipid metabolism in women with polycystic ovary syndrome: a randomized controlled clinical trial. *Gynecological Endocrinology*.

[46] Tabrizi R., Ostadmohammadi V., Lankarani K. B. (2018). The effects of inositol supplementation on lipid profiles among patients with metabolic diseases: a systematic review and meta-analysis of randomized controlled trials. *Lipids in Health and Disease*.

[47] Gao Y. F., Zhang M. N., Wang T. X., Wu T. C., Ai R. D., Zhang Z. S. (2016). Hypoglycemic effect of D-chiro-inositol in type 2 diabetes mellitus rats through the PI3K/Akt signaling pathway. *Molecular and Cellular Endocrinology*.

